# (3aR*,7aS*)-1-(*p*-Tolyl­sulfon­yl)perhydro­indol-2-one

**DOI:** 10.1107/S1600536810013139

**Published:** 2010-04-17

**Authors:** Lei Fang, Xu-Bin Fang, Lei Chen

**Affiliations:** a Department of Chemistry and Chemical Engineering, Southeast University, Nanjing 211189, People’s Republic of China

## Abstract

In the racemic title compound, C_15_H_19_NO_3_S, the dihedral angle between the planes of the benzene ring and the O=S=O group is 56.92 (7)° and the cyclo­hexane ring adopts a chair conformation.

## Related literature

For related structures, see: Brion *et al.* (1992 ▶). For the medicinal background, see: De Ponti *et al.* (1991 ▶).
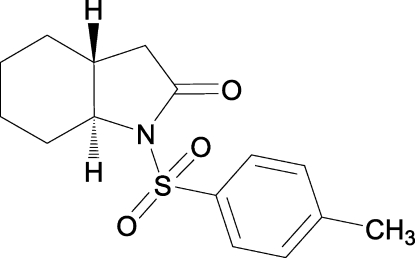


## Experimental

### 

#### Crystal data

C_15_H_19_NO_3_S
*M* *_r_* = 293.37
Orthorhombic, 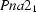

*a* = 15.6509 (12) Å
*b* = 5.9692 (5) Å
*c* = 15.7967 (13) Å
*V* = 1475.8 (2) Å^3^
*Z* = 4
Mo *K*α radiation
μ = 0.23 mm^−1^
*T* = 120 K
0.25 × 0.20 × 0.18 mm

#### Data collection

Bruker SMART CCD diffractometer
Absorption correction: multi-scan (*SADABS*; Bruker, 2000 ▶) *T*_min_ = 0.946, *T*_max_ = 0.961
7103 measured reflections
2702 independent reflections
2397 reflections with *I* > 2σ(*I*)
*R*_int_ = 0.024

#### Refinement

*R*[*F* ^2^ > 2σ(*F* ^2^)] = 0.039
*wR*(*F* ^2^) = 0.102
*S* = 1.01
2702 reflections
182 parameters
1 restraint
H-atom parameters constrained
Δρ_max_ = 0.24 e Å^−3^
Δρ_min_ = −0.17 e Å^−3^
Absolute structure: Flack (1983 ▶), 1189 Friedel pairs
Flack parameter: 0.01 (10)

### 

Data collection: *SMART* (Bruker, 2000 ▶); cell refinement: *SAINT* (Bruker, 2000 ▶); data reduction: *SAINT*; program(s) used to solve structure: *SHELXTL* (Sheldrick, 2008 ▶); program(s) used to refine structure: *SHELXTL*; molecular graphics: *SHELXTL*; software used to prepare material for publication: *SHELXTL*.

## Supplementary Material

Crystal structure: contains datablocks I, global. DOI: 10.1107/S1600536810013139/hb5400sup1.cif

Structure factors: contains datablocks I. DOI: 10.1107/S1600536810013139/hb5400Isup2.hkl

Additional supplementary materials:  crystallographic information; 3D view; checkCIF report

## Figures and Tables

**Fig. 1.**
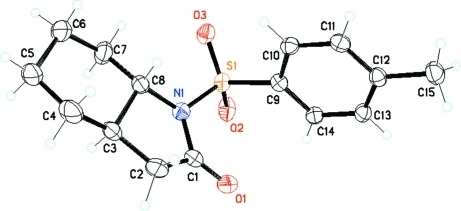
Structure of (I) with 30% displacement ellipsoids.

## supplementary crystallographic information

### Comment

Trandolapril, a potent angiotensin-converting enzyme (ACE) inhibitor, has been widely used for the treatment of hypertension (De Ponti *et al.*, 1991). However, its synthesis procedure is relatively complicated, especially to construct the stereochemical centers of the molecule. To solve the problem, many methods have been proposed in the past years (Brion *et al.*, 1992). Introducing chiral auxiliary-induced stereoselective groups is one of the most promising synthetic strategies since it requires fewer reactions steps and lead to high enantioselectivity. Currently, using *p*-toluenesulfonyl group as stereoselectivity-inducing group, we have successfully synthesized the title compound as a key intermediate for the synthesis of trandolapril.

In the compound, the S=O distances are 1.4261 (19) and 1.426 (2) Å, and the angle of O=S=O is 119.20 (12)deg. The angle of the benzene ring and the plane of O=S=O is 56.92 (7) deg. Meanwhile, the cyclohexane portion adpots a chair structure.

### Experimental

Chloramine-T (2.3 g, 10 mmol) was reacted with iodine (0.2 g, 1 mmol) and cyclohexene (2.05 g, 25 mmol) in acetonitrile (15 ml) for 19 h at room temperature to give *N*-(*p*-toluenesulfonyl)-[b,c] -cyclohexeneaziridine-1*H*-indole-2-one in a yield of 86%. The crude product was directly treated with diethylmalonate (2.4 g, 15 mmol) and sodium ethoxide (1 g, 15 mmol) in THF at room temperature, offering (3a*R*, 7a*S*)-*N* -(*p*-toluenesulfonyl)- 3-ethoxycarbonyloctahydro-1*H*-indole-2-one in a yield of 70%. The obtained compound, together with water (0.2 ml) and sodium chloride (0.35 g), was then dissolved in DMF and warmed to 145 deg for 18 h to yield the title compound in 65% yield as colourless blocks.

### Refinement

All the H atoms were positioned geometrically and refined using a riding model with C—H = 0.95-1.00 Å, and with *U*_iso_(H) = 1.5 for the H atoms of methyl group and 1.2 *U*_iso_(C) for other H atoms.

### Crystal data

**Table:**

C_15_H_19_NO_3_S	*F*(000) = 624
*M**_r_* = 293.37	*D*_x_ = 1.320 Mg m^−^^3^
Orthorhombic, *P**n**a*2_1_	Mo *K*α radiation, λ = 0.71073 Å
Hall symbol: P 2c -2n	Cell parameters from 2285 reflections
*a* = 15.6509 (12) Å	θ = 2.6–25.2°
*b* = 5.9692 (5) Å	µ = 0.23 mm^−^^1^
*c* = 15.7967 (13) Å	*T* = 120 K
*V* = 1475.8 (2) Å^3^	Block, colorless
*Z* = 4	0.25 × 0.20 × 0.18 mm

### Data collection

**Table:**

Bruker SMART CCD diffractometer	2702 independent reflections
Radiation source: fine-focus sealed tube	2397 reflections with *I* > 2σ(*I*)
graphite	*R*_int_ = 0.024
φ and ω scan	θ_max_ = 26.0°, θ_min_ = 2.6°
Absorption correction: multi-scan (*SADABS*; Bruker, 2000)	*h* = −19→19
*T*_min_ = 0.946, *T*_max_ = 0.961	*k* = −7→7
7103 measured reflections	*l* = −19→19

### Refinement

**Table:**

Refinement on *F*^2^	Secondary atom site location: difference Fourier map
Least-squares matrix: full	Hydrogen site location: inferred from neighbouring sites
*R*[*F*^2^ > 2σ(*F*^2^)] = 0.039	H-atom parameters constrained
*wR*(*F*^2^) = 0.102	*w* = 1/[σ^2^(*F*_o_^2^) + (0.0603*P*)^2^ + 0.1589*P*] where *P* = (*F*_o_^2^ + 2*F*_c_^2^)/3
*S* = 1.01	(Δ/σ)_max_ = 0.001
2702 reflections	Δρ_max_ = 0.24 e Å^−^^3^
182 parameters	Δρ_min_ = −0.17 e Å^−^^3^
1 restraint	Absolute structure: Flack (1983), 1189 Friedel pairs
Primary atom site location: structure-invariant direct methods	Flack parameter: 0.01 (10)

### Special details

**Table:**

Geometry. All esds (except the esd in the dihedral angle between two l.s. planes) are estimated using the full covariance matrix. The cell esds are taken into account individually in the estimation of esds in distances, angles and torsion angles; correlations between esds in cell parameters are only used when they are defined by crystal symmetry. An approximate (isotropic) treatment of cell esds is used for estimating esds involving l.s. planes.
Refinement. Refinement of *F*^2^ against ALL reflections. The weighted *R*-factor wR and goodness of fit *S* are based on *F*^2^, conventional *R*-factors *R* are based on *F*, with *F* set to zero for negative *F*^2^. The threshold expression of *F*^2^ > σ(*F*^2^) is used only for calculating *R*-factors(gt) etc. and is not relevant to the choice of reflections for refinement. *R*-factors based on *F*^2^ are statistically about twice as large as those based on *F*, and *R*- factors based on ALL data will be even larger.

### Fractional atomic coordinates and isotropic or equivalent isotropic displacement parameters (Å^2^)

**Table:**

	*x*	*y*	*z*	*U*_iso_*/*U*_eq_	
S1	−0.00414 (4)	0.18513 (9)	0.40616 (5)	0.03841 (17)	
C1	−0.0558 (2)	0.0780 (5)	0.56190 (17)	0.0516 (7)	
C2	−0.0387 (2)	−0.1072 (6)	0.62443 (18)	0.0629 (9)	
H2A	−0.0509	−0.0577	0.6830	0.075*	
H2B	−0.0737	−0.2410	0.6116	0.075*	
C3	0.0549 (2)	−0.1554 (5)	0.61272 (16)	0.0489 (7)	
H3A	0.0873	−0.0347	0.6426	0.059*	
C4	0.0933 (2)	−0.3773 (5)	0.63948 (19)	0.0663 (9)	
H4A	0.0644	−0.5014	0.6093	0.080*	
H4B	0.0850	−0.3995	0.7010	0.080*	
C5	0.1881 (2)	−0.3783 (6)	0.61869 (18)	0.0703 (10)	
H5A	0.2118	−0.5287	0.6307	0.084*	
H5B	0.2178	−0.2694	0.6557	0.084*	
C6	0.2056 (2)	−0.3187 (6)	0.5266 (2)	0.0691 (10)	
H6A	0.1856	−0.4431	0.4903	0.083*	
H6B	0.2681	−0.3043	0.5185	0.083*	
C7	0.16246 (19)	−0.1007 (5)	0.49752 (18)	0.0569 (8)	
H7A	0.1872	0.0292	0.5277	0.068*	
H7B	0.1708	−0.0789	0.4360	0.068*	
C8	0.06903 (17)	−0.1222 (4)	0.51752 (16)	0.0395 (6)	
H8A	0.0461	−0.2562	0.4871	0.047*	
C9	−0.10350 (16)	0.0904 (4)	0.36999 (14)	0.0342 (5)	
C10	−0.10958 (16)	−0.1217 (4)	0.33444 (15)	0.0376 (5)	
H10A	−0.0614	−0.2183	0.3332	0.045*	
C11	−0.18653 (18)	−0.1900 (4)	0.30096 (15)	0.0405 (6)	
H11A	−0.1912	−0.3357	0.2773	0.049*	
C12	−0.25761 (16)	−0.0497 (4)	0.30111 (15)	0.0391 (6)	
C13	−0.25008 (16)	0.1610 (4)	0.33775 (17)	0.0403 (6)	
H13A	−0.2981	0.2585	0.3385	0.048*	
C14	−0.17402 (17)	0.2307 (4)	0.37301 (15)	0.0374 (6)	
H14A	−0.1700	0.3737	0.3991	0.045*	
C15	−0.3410 (2)	−0.1241 (5)	0.26208 (19)	0.0546 (7)	
H15A	−0.3581	−0.2681	0.2866	0.066*	
H15B	−0.3337	−0.1408	0.2008	0.066*	
H15C	−0.3852	−0.0119	0.2735	0.066*	
N1	0.01047 (13)	0.0708 (3)	0.50183 (13)	0.0401 (5)	
O1	−0.11421 (15)	0.2106 (4)	0.55905 (13)	0.0689 (6)	
O2	−0.00807 (12)	0.4227 (3)	0.41542 (15)	0.0541 (6)	
O3	0.06024 (11)	0.0901 (3)	0.35308 (11)	0.0479 (5)	

### Atomic displacement parameters (Å^2^)

**Table:**

	*U*^11^	*U*^22^	*U*^33^	*U*^12^	*U*^13^	*U*^23^
S1	0.0388 (4)	0.0307 (3)	0.0457 (3)	−0.0040 (2)	−0.0067 (3)	0.0048 (3)
C1	0.0526 (19)	0.0666 (18)	0.0355 (12)	0.0096 (15)	−0.0052 (12)	−0.0115 (13)
C2	0.064 (2)	0.089 (2)	0.0359 (13)	0.0132 (19)	0.0076 (14)	0.0059 (14)
C3	0.060 (2)	0.0554 (16)	0.0316 (11)	0.0074 (14)	−0.0060 (12)	−0.0037 (11)
C4	0.096 (3)	0.0637 (19)	0.0396 (15)	0.0170 (18)	−0.0036 (16)	0.0128 (13)
C5	0.089 (3)	0.079 (2)	0.0424 (15)	0.038 (2)	−0.0088 (16)	0.0009 (14)
C6	0.073 (2)	0.088 (2)	0.0465 (16)	0.0378 (19)	−0.0027 (16)	0.0060 (15)
C7	0.046 (2)	0.0692 (19)	0.0554 (17)	0.0109 (14)	−0.0035 (14)	0.0126 (15)
C8	0.0482 (16)	0.0333 (12)	0.0370 (12)	0.0049 (11)	−0.0065 (11)	−0.0023 (10)
C9	0.0394 (15)	0.0287 (11)	0.0346 (11)	−0.0015 (9)	−0.0004 (11)	0.0042 (9)
C10	0.0437 (16)	0.0320 (12)	0.0370 (11)	0.0077 (10)	0.0004 (12)	0.0007 (10)
C11	0.0502 (17)	0.0343 (13)	0.0370 (12)	−0.0016 (11)	−0.0008 (11)	−0.0045 (9)
C12	0.0418 (16)	0.0415 (13)	0.0340 (11)	−0.0054 (11)	−0.0030 (10)	0.0033 (10)
C13	0.0375 (16)	0.0408 (13)	0.0425 (12)	0.0045 (10)	−0.0036 (11)	0.0005 (10)
C14	0.0412 (16)	0.0298 (11)	0.0414 (11)	0.0022 (10)	−0.0015 (11)	−0.0017 (10)
C15	0.047 (2)	0.0621 (18)	0.0543 (16)	−0.0116 (14)	−0.0083 (13)	−0.0042 (14)
N1	0.0433 (14)	0.0376 (12)	0.0394 (11)	0.0038 (9)	−0.0038 (9)	−0.0045 (9)
O1	0.0639 (15)	0.0967 (17)	0.0462 (11)	0.0313 (13)	−0.0031 (10)	−0.0131 (11)
O2	0.0524 (13)	0.0302 (9)	0.0797 (15)	−0.0061 (8)	−0.0220 (11)	0.0006 (11)
O3	0.0392 (11)	0.0581 (11)	0.0463 (10)	−0.0009 (8)	0.0028 (8)	0.0126 (8)

### Geometric parameters (Å, °)

**Table:**

S1—O2	1.4267 (17)	C6—H6B	0.9900
S1—O3	1.4281 (19)	C7—C8	1.502 (4)
S1—N1	1.674 (2)	C7—H7A	0.9900
S1—C9	1.750 (2)	C7—H7B	0.9900
C1—O1	1.210 (3)	C8—N1	1.493 (3)
C1—N1	1.406 (4)	C8—H8A	1.0000
C1—C2	1.507 (4)	C9—C14	1.386 (3)
C2—C3	1.505 (5)	C9—C10	1.388 (3)
C2—H2A	0.9900	C10—C11	1.377 (4)
C2—H2B	0.9900	C10—H10A	0.9500
C3—C4	1.514 (4)	C11—C12	1.392 (4)
C3—C8	1.533 (3)	C11—H11A	0.9500
C3—H3A	1.0000	C12—C13	1.389 (3)
C4—C5	1.520 (5)	C12—C15	1.510 (4)
C4—H4A	0.9900	C13—C14	1.379 (3)
C4—H4B	0.9900	C13—H13A	0.9500
C5—C6	1.522 (4)	C14—H14A	0.9500
C5—H5A	0.9900	C15—H15A	0.9800
C5—H5B	0.9900	C15—H15B	0.9800
C6—C7	1.537 (4)	C15—H15C	0.9800
C6—H6A	0.9900		
			
O2—S1—O3	119.03 (12)	C8—C7—C6	107.0 (3)
O2—S1—N1	108.56 (11)	C8—C7—H7A	110.3
O3—S1—N1	105.77 (10)	C6—C7—H7A	110.3
O2—S1—C9	108.42 (10)	C8—C7—H7B	110.3
O3—S1—C9	107.88 (12)	C6—C7—H7B	110.3
N1—S1—C9	106.52 (11)	H7A—C7—H7B	108.6
O1—C1—N1	123.5 (3)	N1—C8—C7	119.8 (2)
O1—C1—C2	129.7 (3)	N1—C8—C3	100.0 (2)
N1—C1—C2	106.8 (2)	C7—C8—C3	111.0 (2)
C3—C2—C1	103.5 (2)	N1—C8—H8A	108.5
C3—C2—H2A	111.1	C7—C8—H8A	108.5
C1—C2—H2A	111.1	C3—C8—H8A	108.5
C3—C2—H2B	111.1	C14—C9—C10	120.7 (2)
C1—C2—H2B	111.1	C14—C9—S1	120.07 (17)
H2A—C2—H2B	109.0	C10—C9—S1	119.17 (18)
C2—C3—C4	121.3 (3)	C11—C10—C9	119.0 (2)
C2—C3—C8	103.7 (2)	C11—C10—H10A	120.5
C4—C3—C8	109.3 (2)	C9—C10—H10A	120.5
C2—C3—H3A	107.3	C10—C11—C12	121.3 (2)
C4—C3—H3A	107.3	C10—C11—H11A	119.3
C8—C3—H3A	107.3	C12—C11—H11A	119.3
C3—C4—C5	109.3 (3)	C13—C12—C11	118.5 (2)
C3—C4—H4A	109.8	C13—C12—C15	120.7 (2)
C5—C4—H4A	109.8	C11—C12—C15	120.8 (2)
C3—C4—H4B	109.8	C14—C13—C12	121.0 (2)
C5—C4—H4B	109.8	C14—C13—H13A	119.5
H4A—C4—H4B	108.3	C12—C13—H13A	119.5
C4—C5—C6	112.4 (3)	C13—C14—C9	119.4 (2)
C4—C5—H5A	109.1	C13—C14—H14A	120.3
C6—C5—H5A	109.1	C9—C14—H14A	120.3
C4—C5—H5B	109.1	C12—C15—H15A	109.5
C6—C5—H5B	109.1	C12—C15—H15B	109.5
H5A—C5—H5B	107.9	H15A—C15—H15B	109.5
C5—C6—C7	113.8 (2)	C12—C15—H15C	109.5
C5—C6—H6A	108.8	H15A—C15—H15C	109.5
C7—C6—H6A	108.8	H15B—C15—H15C	109.5
C5—C6—H6B	108.8	C1—N1—C8	111.4 (2)
C7—C6—H6B	108.8	C1—N1—S1	119.73 (18)
H6A—C6—H6B	107.7	C8—N1—S1	123.25 (16)
